# Supplementary figures and images for: Adaptation mechanisms of Listeria monocytogenes to quaternary ammonium compounds
Source: Microbiol Spectr. 2023 Sep 11;11(5):e01441-23. doi: 10.1128/spectrum.01441-23 (PMC10580936; doi:10.1128/spectrum.01441-23)

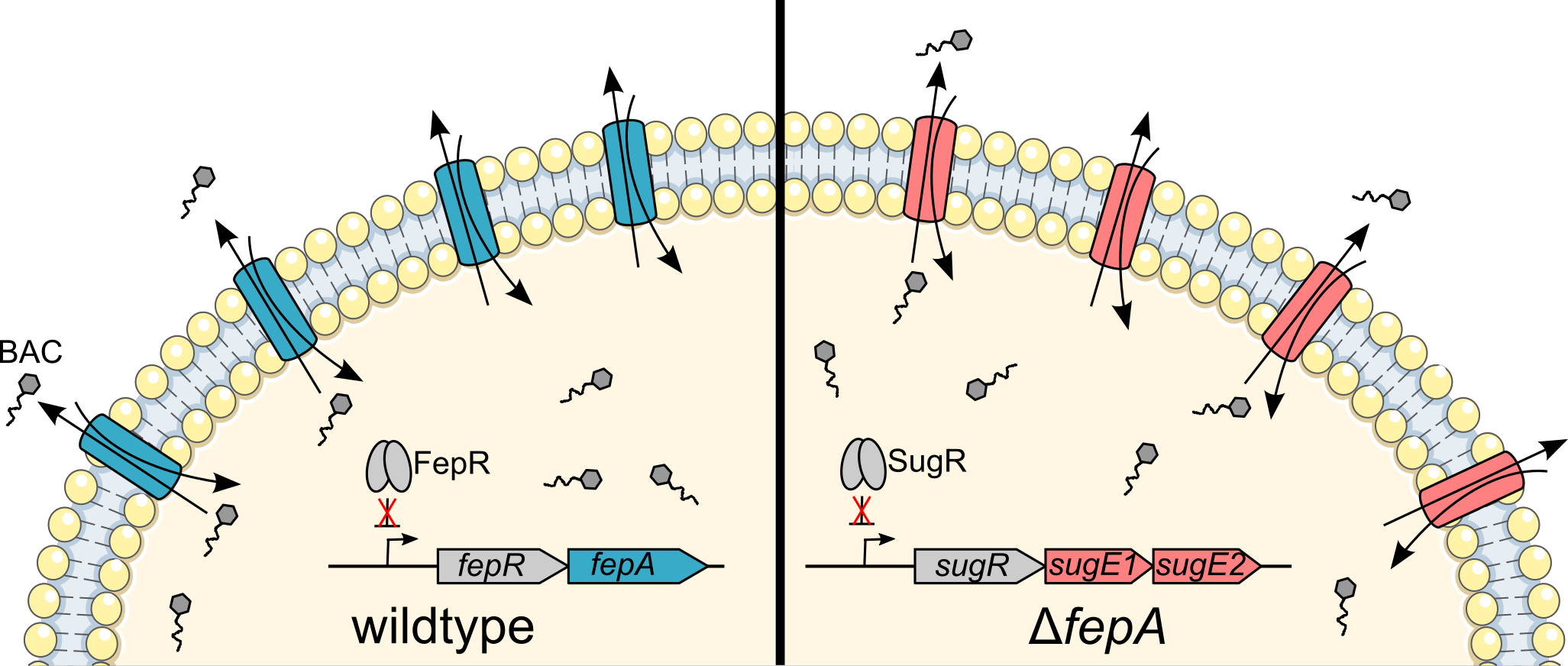

Supplement: Supplemental Image — Graphical abstract. [file spectrum.01441-23-s0001.tif]
