# Supplementary material for: Adaptation mechanisms of Listeria monocytogenes to quaternary ammonium compounds
Source: Microbiol Spectr. 2023 Sep 11;11(5):e01441-23. doi: 10.1128/spectrum.01441-23 (PMC10580936; doi:10.1128/spectrum.01441-23)
Supplement: Supplementary Material 1 — Table S1 and S2; Figures S1 and S2. [file spectrum.01441-23-s0002.docx]

**Supplemental Material**

**Adaptation mechanisms of *Listeria monocytogenes* to quaternary ammonium compounds**

Lisa Maria Schulz, Fabienne Dreier, Lisa Marie de Sousa Miranda, Jeanine Rismondo^*^

Department of General Microbiology, Institute of Microbiology and Genetics, GZMB, Georg-August University Göttingen, Grisebachstr. 8, 37077 Göttingen, Germany

* Correspondence: jrismon@gwdg.de

**Running title:** Adaptation of *L. monocytogenes* to biocides

**Supplemental Tables**

**Table S1: Bacterial strains used in this study**

| Unique ID | Strain name and resistance | Source |
| --- | --- | --- |
| *Escherichia coli* strains | | |
| ANG124 | DH5α pKSV7; AmpR | (1) |
| ANG4243 | XL1-Blue pIMK3; KanR | (2) |
| ANG5181 | XL1-Blue pPL3e-*lacZ*; CamR | (3) |
| EJR149 | XL10-Gold pWH844; AmpR | (4) |
| EJR227 | XL10-Gold pIMK3-*fepA*; KanR | This study |
| EJR229 | XL1-Blue pKSV7-Δ*sugE1/2*; AmpR | This study |
| EJR230 | XL1-Blue pKSV7-Δ*fepA*; AmpR | This study |
| EJR242 | XL10-Gold pWH844-*fepR*; AmpR | This study |
| EJR248 | XL10-Gold pWH844-*fepR^L24F^*; AmpR | This study |
| EJR257 | DH5α pPL3e-*P_fepR_*-*lacZ*; CamR | This study |
| EJR258 | DH5α pPL3e-*P_fepR_^A-33G^*-*lacZ*; CamR | This study |
| EJR259 | XL10-Gold pIMK3-*sugE1/2*; KanR | This study |
| EJR260 | DH5α pPL3e-*P_fepR_^G-27T^*-*lacZ*; CamR | This study |
| *Listeria monocytogenes* strains | | |
| ANG873 | EGD-e | (5) |
| LJR187 | EGD-e *fepR^N170fs^* | This study |
| LJR188 | EGD-e *P_fepR_*^G-27T^ | This study |
| LJR190 | EGD-e *fepR^G157*^* | This study |
| LJR194 | EGD-e *fepR*^Δ^*^45-46^* | This study |
| LJR196 | EGD-e *fepR*^Δ^*^99^* | This study |
| LJR208 | EGD-e *fepR^Q140*^* | This study |
| LJR209 | EGD-e *fepR^Y155*^* | This study |
| LJR210 | EGD-e *fepR^Q140*^* | This study |
| LJR211 | EGD-e *fepR^V115D^* | This study |
| LJR212 | EGD-e *fepR*^Δ^*^45-46^* | This study |
| LJR213 | EGD-e *fepR^S23L^* | This study |
| LJR214 | EGD-e *fepR^Q140*^* | This study |
| LJR215 | EGD-e *P_fepR_^A-33G^* | This study |
| LJR216 | EGD-e *fepR^M126fs^* | This study |
| LJR217 | EGD-e *fepR*^Δ^*^45-46^* | This study |
| LJR218 | EGD-e *fepR^L24F^* | This study |
| LJR219 | EGD-e *fepR^INS29DIA^* | This study |
| LJR220 | EGD-e *fepR*^Δ^*^45-46^* | This study |
| LJR221 | EGD-e *fepR^W137fs^* | This study |
| LJR222 | EGD-e *fepR^M126fs^* | This study |
| LJR231 | EGD-e pIMK3-*fepA*; KanR | This study |
| LJR234 | EGD-e *P_sugR_^G-11T^* | This study |
| LJR235 | EGD-e *sugR^F49fs^* | This study |
| LJR248 | EGD-e *sugR^D122*^* | This study |
| LJR249 | EGD-e *sugR^D122*^* | This study |
| LJR250 | EGD-e *sugR^D122*^* | This study |
| LJR257 | EGD-e *sugR^L64*^* | This study |
| LJR258 | EGD-e *sugR^F49fs^* | This study |
| LJR259 | EGD-e *sugR^F49fs^* | This study |
| LJR260 | EGD-e *sugR^F49fs^* | This study |
| LJR261 | EGD-e Δ*fepA* | This study |
| LJR262 | EGD-e Δ*sugE1/2* | This study |
| LJR265 | EGD-e Δ*fepA* pIMK3-*fepA*; KanR | This study |
| LJR266 | EGD-e Δ*fepA sugR^D71*^* | This study |
| LJR267 | EGD-e Δ*fepA sugR^F49fs^* | This study |
| LJR268 | EGD-e Δ*fepA sugR^S44*^* | This study |
| LJR269 | EGD-e Δ*fepA sugR^A23D^* | This study |
| LJR270 | EGD-e Δ*sugE1/2 fepR^G157*^* | This study |
| LJR271 | EGD-e Δ*sugE1/2 fepR^I185fs^* | This study |
| LJR272 | EGD-e Δ*sugE1/2 fepR^D171fs^* | This study |
| LJR273 | EGD-e Δ*sugE1/2 fepR^P107L^* | This study |
| LJR274 | EGD-e Δ*sugE1/2 fepR^E89fs^* | This study |
| LJR275 | EGD-e Δ*sugE1/2 fepR^A154E^* | This study |
| LJR276 | EGD-e Δ*sugE1/2 fepR^V115fs^* | This study |
| LJR277 | EGD-e Δ*sugE1/2 fepR^V115fs^* | This study |
| LJR280 | EGD-e Δ*fepA sugR^L57*^* | This study |
| LJR281 | EGD-e Δ*fepA sugR^Y19fs^* | This study |
| LJR282 | EGD-e Δ*fepA sugR^S81*^* | This study |
| LJR283 | EGD-e Δ*fepA sugR^S81*^* | This study |
| LJR301 | EGD-e pIMK3-*sugE1/2*; KanR | This study |
| LJR302 | EGD-e pPL3e-*P_fepR_^A-33G^*-*lacZ*; ErmR | This study |
| LJR303 | EGD-e pPL3e-*P_fepR_^G-27T^*-*lacZ*; ErmR | This study |
| LJR326 | EGD-e Δ*fepA*Δ*sugE1/2 lmo1753^K19fs^,* short: Δ*fepA*Δ*sugE1/2* BAC1 | This study |
| LJR327 | EGD-e Δ*fepA*Δ*sugE1/2 lmo1753^K19fs^,* short: Δ*fepA*Δ*sugE1/2* CTAB1 | This study |
| LJR328 | EGD-e Δ*fepA*Δ*sugE1/2 lmo1753^K19fs^,* short: Δ*fepA*Δ*sugE1/2* CTAB2 | This study |
| LJR329 | EGD-e Δ*fepA*Δ*sugE1/2* | This study |
| LJR330 | EGD-e Δ*fepA*Δ*sugE1/2 lmo1753^V225fs^ P_lmo1682_^G-37A^,* short: Δ*fepA*Δ*sugE1/2* BAC2 | This study |
| LJR336 | EGD-e pPL3e-*P_fepR_*-*lacZ*; ErmR | This study |

fs – frameshift

**Table S2: Primers used in this study**

| **Number** | **Name** | **Sequence** |
| --- | --- | --- |
| FD1 | pWH844-*fepR* fw | AAAGGATCCAGAAAAGAAGAAATCAAACAAGCTGC |
| FD2 | pWH844-*fepR* rev | AAAGTCGACTTAATTCAAAGCTTTTAGCGTAATTCCTCTC |
| FD3 | *P_fepR_* fw | GACATACGAATTGATTAGCGAATTTTTAGAA |
| FD4 | *P_fepR_* rev | CATTCCACTCCTCTCACAAAAACTG |
| FD5 | pPL3e-*P_fepR_* fw | AAAGGATCCGACATACGAATTGATTAGCGAATTTTTAGA |
| FD6 | pPL3e-*P_fepR_* rev | AAAGTCGACTTCTTTTCTCATCATTCCACTCCTCT |
| JR247 | *sugE1/2* up fw | ACGCGTCGACGGCAGAACTAGTTAATGAGAAG |
| JR248 | *sugE1/2* up rev | TTTCAATCCGACCCCTGCCATAATCAAATAAAACC |
| JR249 | *sugE1/2* down fw | ATTATGGCAGGGGTCGGATTGAAATTAACATCTGG |
| JR250 | *sugE1/2* down rev | CGGGGTACCGGCAACTGCACCTTCTGG |
| JR262 | pIMK3-*sugE1/2* fw | CATGCCATGGGGGCTTGGTTTTATTTGATTATGGCAG |
| JR263 | pIMK3-*sugE1/2* rev | ACGCGTCGACTTAAACGCCAGATGTTAATTTCAATC |
| LMS478 | pIMK3-*fepA* fw | AAACCATGGGGGCAAAAAATATGGAAATTTTAGAAACAGATTCA |
| LMS479 | pIMK3-*fepA* rev | TTTGTCGACTTATTTAAATAAAATATGTTTTTTCTTCATATAGAATACAATG |
| LMS484 | *fepA* up rev | TCGTTTTTATTTTGTTTCTAAAATTTCCATATTTTTTGCCATACTA |
| LMS485 | *fepA* up fw | AAAGTCGACACCAATACGTAGCAAAGATTTAGTCG |
| LMS486 | *fepA* down fw | ATTTTAGAAACAAAATAAAAACGAGACGAAGATAGATGATATC |
| LMS487 | *fepA* down rev | TTTGGTACCGCCACCTGTAAACAATAAAAGCTAAG |

**Supplemental Figures**


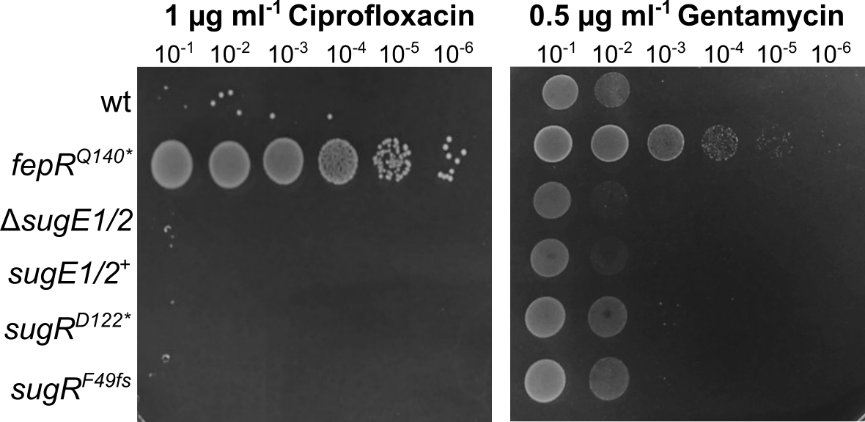


**Figure S1: Cross-resistance of *sugR* mutant strains**

Drop dilution assays of *L. monocytogenes* strains EGD-e (wt), a *sugE1/2* deletion strain (Δ*sugE1/2*), a wt strain containing the IPTG-inducible pIMK3-*sugE1/2* plasmid LJR301 (*sugE1/2*^+^) and the suppressor mutants *sugR^D122*^* (LJR248), and *sugR^F49fs*^* (LJR258). The *fepR^Q140*^* suppressor mutant was used as a control. Cells were propagated on BHI plates or BHI plates containing 1 µg ml^-1^ ciprofloxacin or 0.5 µg ml^-1^ gentamycin. All plates were supplemented with 1 mM IPTG to induce the expression of *sugE1/2* in the *sugE1/E2*^+^ strain and plates were incubated overnight at 37°C. A representative image of at least three biological replicates is shown.


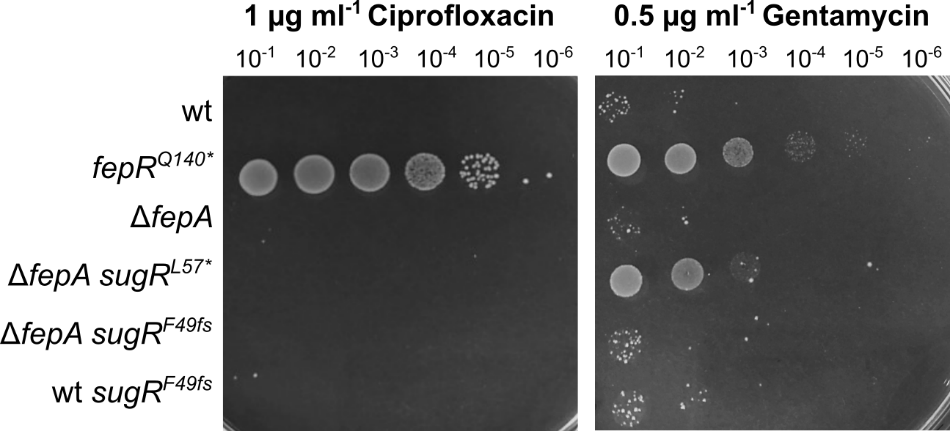


**Figure S2: Cross-resistance of Δ*fepA* *sugR* mutant strains**

Drop dilution assays of *L. monocytogenes* strains EGD-e (wt), the *fepA* deletion strain (Δ*fepA*) and the suppressor mutants Δ*fepA sugR^L57^*^*^(LJR280), Δ*fepA sugR^F49fs^* (LJR267) and wt *sugR^F49fs^* (LJR258). The *fepR^Q140*^* suppressor mutant (LJR208) was used as a control. Cells were propagated on BHI plates or BHI plates containing 1 µg ml^-1^ ciprofloxacin or 0.5 µg ml^-1^ gentamycin and were incubated overnight at 37°C. A representative image of at least three biological replicates is shown.

**References**

1. Smith K, Youngman P. 1992. Use of a new integrational vector to investigate compartment-specific expression of the *Bacillus subtilis* *spoIIM* gene. Biochimie 74:705–711. doi:10.1016/0300-9084(92)90143-3.

2. Monk IR, Gahan CGM, Hill C. 2008. Tools for functional postgenomic analysis of *Listeria monocytogenes*. Appl Environ Microbiol 74:3921–3934. doi:10.1128/AEM.00314-08.

3. Rismondo J, Halbedel S, Gründling A. 2019. Cell shape and antibiotic resistance are maintained by the activity of multiple FtsW and RodA enzymes in *Listeria monocytogenes*. mBio 10. doi:10.1128/mBio.01448-19.

4. Schirmer F, Ehrt S, Hillen W. 1997. Expression, inducer spectrum, domain structure, and function of MopR, the regulator of phenol degradation in *Acinetobacter calcoaceticus* NCIB8250. J Bacteriol 179:1329–1336. doi:10.1128/jb.179.4.1329-1336.1997.

5. Glaser P, Frangeul L, Buchrieser C, Rusniok C, Amend A, Baquero F, Berche P, Bloecker H, Brandt P, Chakraborty T, Charbit A, Chetouani F, Couvé E, Daruvar A de, Dehoux P, Domann E, Domínguez-Bernal G, Duchaud E, Durant L, Dussurget O, Entian KD, Fsihi H, García-del Portillo F, Garrido P, Gautier L, Goebel W, Gómez-López N, Hain T, Hauf J, Jackson D, Jones LM, Kaerst U, Kreft J, Kuhn M, Kunst F, Kurapkat G, Madueno E, Maitournam A, Vicente JM, Ng E, Nedjari H, Nordsiek G, Novella S, Pablos B de, Pérez-Diaz JC, Purcell R, Remmel B, Rose M, Schlueter T, Simoes N, Tierrez A, Vázquez-Boland JA, Voss H, Wehland J, Cossart P. 2001. Comparative genomics of *Listeria* species. Science 294:849–852. doi:10.1126/science.1063447.
